# Ensemble learning for predicting ex vivo human placental barrier permeability

**DOI:** 10.1186/s12859-022-04937-y

**Published:** 2022-09-22

**Authors:** Che-Yu Chou, Pinpin Lin, Jongwoon Kim, Shan-Shan Wang, Chia-Chi Wang, Chun-Wei Tung

**Affiliations:** 1 Graduate Institute of Data Science, Taipei Medical University, Taipei, Taiwan; 2 National Institute of Environmental Health Sciences, National Health Research Institutes, Miaoli County, Taiwan; 3 Chemical Safety Research Center, Korea Research Institute of Chemical Technology (KRICT), Daejeon, Republic of Korea; 4 Department and Graduate Institute of Veterinary Medicine, School of Veterinary Medicine, National Taiwan University, Taipei, Taiwan; 5 Institute of Biotechnology and Pharmaceutical Research, National Health Research Institutes, Miaoli County, Taiwan

**Keywords:** Machine learning, Ensemble learning, Alternative method, Placental barrier permeability

## Abstract

**Background:**

The placental barrier protects the fetus from exposure to some toxicants and is vital for drug development and risk assessment of environmental chemicals. However, in vivo experiments for assessing the placental barrier permeability of chemicals is not ethically acceptable. Although ex vivo placental perfusion methods provide good alternatives for the assessment of placental barrier permeability, the application to a large number of test chemicals could be time- and resource-consuming. Computational prediction models for ex vivo placental barrier permeability are therefore desirable.

**Methods:**

A total of 87 chemicals and corresponding 1444 physicochemical properties were divided into training and test datasets. Three types of algorithms including linear regression, random forest, and ensemble models were applied to develop prediction models for ex vivo placental barrier permeability.

**Results:**

Among the tested models, the ensemble model integrating the previous two methods performed best for predicting ex vivo human placental barrier permeability with correlation coefficients of 0.887 and 0.825 when considering the applicability domain. An additional test on seven newly curated chemicals from the literature showed a good correlation coefficient of 0.879 which was further improved to 0.921 by considering the variation of experiments.

**Conclusion:**

In this study, the first valid predicting model for ex vivo human placental barrier permeability was developed following the OECD guideline. The model is expected to be useful for assessing the human placental barrier permeability and can be integrated with developmental toxicity prediction models for investigating the toxic effects of chemicals on the fetus.

**Supplementary Information:**

The online version contains supplementary material available at 10.1186/s12859-022-04937-y.

## Background

The exposure of certain chemical compounds may cause toxic effects in the human body. Exposure sources of the toxic chemicals could be found in the environment, food, or drugs in our daily life. While a low dose of toxic chemicals may not cause adverse effects in adults, chemicals capable of penetrating the placental barrier could be harmful to the fetus which is a significant problem. Due to the complexity and ethical issues, the measurement of placental transfer permeability of chemicals based on in vivo conditions may not be applicable for risk assessment of novel chemicals. Therefore, a few computational methods have been developed to predict in vivo human fetal-maternal blood concentration ratio (logFM) of chemicals [1, 2]. Takaku et al. conducted the first work on the prediction model for predicting in vivo logFM values based on manually curated 55 chemicals with logFM values [2]. Subsequently, Wang et al. proposed the first valid model according to the OECD guideline with fewer features but higher performance [1].

While the above-mentioned prediction models could be useful for studying in vivo human placental barrier permeability, the in vivo human dataset used for training the models was quite small and it is not likely to grow due to ethical issues. Further improvement and validation of the models will be infeasible without the input of new in vivo data. Since the placenta is the most species-specific mammalian organ, the adaptation of animal data for extrapolation to human beings has not been applicable [3].

The integration of multiple relevant models could largely improve the prediction performance and enhance regulatory acceptance. For example, assays regarding the adverse outcome pathway of skin sensitization have been integrated for developing novel prediction models [4–6]. The prediction of developmental and reproductive toxicity can be improved by a weight-of-evidence integration of several types of models [7]. For ICH (International Council for Harmonisation of Technical Requirements for Pharmaceuticals for Human Use) M7 guidelines, an agreement of mutagenicity prediction outcomes from two complementary models, e.g. rule-based and statistical models, is acceptable for regulation [8]. Altogether, it is desirable to develop complementary or mechanism-relevant models for human placental barrier permeability.

Ex vivo human placental perfusion models are potential complementary methods. There were two studies aiming to develop prediction models for ex vivo human placental barrier permeability using the clearance index (CI) values, a relative permeability compared to antipyrine. Giaginis et al. applied a multivariate data analysis method incorporating principal component analysis (PCA) and partial least square (PLS) methods to get the most influential variables for model development [9]. Based on 16 descriptors, their model for ex vivo CI values performed reasonably good for CI prediction with fitting and cross-validation r^2^ values of 0.730 and 0.710, respectively. Nevertheless, there is no independent test dataset for evaluating the prediction performance for unseen chemicals, and no applicability domain was defined that is essential according to the OECD guideline for validating quantitative structure–activity relationship models [10]. Zhang et al. identified a different set of 48 descriptors for building a PLS model based on the same dataset [11]. In their work, a test set has been divided from the whole dataset to independently test the developed model. Within their reported applicability domain, r^2^ values are 0.732 and 0.766 for cross-validation on the training dataset and external prediction on the test dataset, respectively. While an applicability domain was defined based on a leverage method [12, 13], the selection of outliers depends on the residues of predicted and experimental values presenting an information leak issue and therefore the testing performance could be overestimated. More importantly, the identification of tested chemicals within the applicability domain based on residues is impractical due to the lack of experimental values of the tested chemicals. Applicability domain should be defined according to the training dataset and tested using the independent test dataset to ensure a proper estimation of prediction performance [1, 4].

To support the identification of potential chemicals permeable to the human placental barrier, this work presents the first valid model according to the OECD guidelines. A sequential forward selection algorithm was applied to select informative features for linear, non-linear, and ensemble models using the training set. Results showed that the proposed ensemble model integrating linear regression and random forest algorithms performed best with correlation coefficients of 0.940, 0.850, and 0.825 for model fitting, leave-one-out cross-validation (LOOCV), and independent test, respectively. After the adjustment of the applicability domain based on only the training set, its performance was improved with correlation coefficients of 0.952 and 0.887 for model fitting and leave-one-out cross-validation (LOOCV). Since all chemicals in the independent test set fall in the applicability domain, there is no change in the independent test performance. In addition, we further collected seven external chemicals with ex vivo permeability data from the literature. A final ensemble model trained using all chemicals in the training and test sets was applied to predict the seven external chemicals. A very good correlation coefficient value of 0.879 was obtained showing the usefulness of the proposed method. By considering the variation of experiments, an excellent correlation coefficient value of 0.921 was obtained. Future works could be the integration of the two models for the consensus prediction of human placental barrier permeability.

## Methods

### Dataset

A total of 88 chemicals with ex vivo human placental barrier permeability data were taken from a previous curation work [11]. Two stereoisomers of dexamethasone and betamethasone were found among the 88 compounds and dexamethasone was removed to avoid overstimulation of the prediction performance. The remaining 87 chemicals were randomly divided into a training dataset and a test dataset with 66 and 21 chemicals, respectively. The permeability data was represented as a clearance index (CI) that is a relative permeability compared to antipyrine designed to overcome inter-placental variability [9] as shown in the following equation Eq. 1.

1 $${\text{CI }} = {\text{ clearance }}\;{\text{of }}\;{\text{drug }}\;{\text{under }}\;{\text{study}}/{\text{clearance}}\;{\text{ of }}\;{\text{antipyrine}}$$

The training dataset was utilized for the development of a final model including feature selection and cross-validation, while the test dataset was used to independently test the final model. Chemicals were firstly converted into 1,444 1D and 2D features (physicochemical properties) using the PaDEL-Descriptor v2.21 software [14] as it showed excellent performance in various tasks [1, 2, 15, 16]. The data tables of training and test datasets are shown in Additional file 1: Tables S1 and S3, respectively.

### Model development and feature selection

Irrelevant features and less informative features with scarcity and small variation can hamper the model performance that should be removed before model development. Three basic steps were firstly applied to exclude useless features with (1) extreme values that are 100-fold larger than the average, (2) more than or equal to 30% zero values (scarcity), and (3) small variation (less than 12 unique values). Before the application of regression algorithms for model development, the remaining features in the training dataset were normalized based on the Z-score method [17] as shown in Eq. 2.

2 $${\text{z }} = \, ({\text{x}} - \mu )/\sigma ,$$

where z, x, μ, and σ are the normalized, original, mean, and variance values, respectively.

Based on the normalized feature vectors, a sequential forward selection algorithm [18] was applied to select informative features based on the performance of leave-one-out cross-validation (LOOCV). Sequential forward/backward selection algorithms are efficient solutions for the combinatorial optimization problems that are highly appreciated in the field of bioinformatics due to the high-dimensional feature space observed in numerous applications [19, 20]. Briefly, the best features were sequentially selected and appended into the final feature set. The selection of features is based on the LOOCV performance of the corresponding regression algorithm.

In this study, we consider three types of algorithms including the linear regression, random forest, and voting regression combining the previous two algorithms. Linear regression presents can capture linear relationships between features and the permeability, however, the oversimplified linear relationships may not be able to fully explain complex biological outpoints. In order to capture non-linear relationships, the popular random forest algorithm [21] was adopted in this study. Since the dataset is small, it is important to avoid overfitting problems. In this study, we argue that predictive features should be useful for both linear and non-linear models. Therefore, a voting regression algorithm combining the linear regression and random forest models was also tested. After the model development, the test dataset was then normalized based on the parameters derived from training the dataset and predicted by the developed models. Scikit-learn package version 0.21 [22] and associated default parameters were utilized to implement the linear regression, random forest, and voting regressor.

### Applicability domain

Applicability domain defines the chemical space that can be reliably predicted by the corresponding model. In this study, a decision tree method [1] was applied to identify the rules for identifying chemicals that may not be reliably predicted by the model. The rules were derived from only the training dataset to avoid information leak issues that might overestimate the prediction performance [1, 4]. We first calculate the absolute error for each chemical based on the LOOCV results as a new dependent variable. Subsequently, the classification and regression tree (CART) algorithm [23] was applied to learn a decision tree from identified informative features for predicting the absolute errors of test chemicals. Once the decision tree has been constructed, *n* rules can be extracted, representing the decision process of *n* corresponding leaf nodes. The rule with a high absolute error indicated the chemical properties involved in the rule could lead to unreliable prediction. Therefore, the identified rules were then ranked based on the absolute error in descending order and iteratively appended to a final rule set until there was no significant performance improvement. The final rule set represents the chemical space that the model may not reliably predict and was utilized for excluding chemicals out of the applicability domain in the test dataset.

## Results

### Comparison of linear and non-linear models

**Fig. 1:**
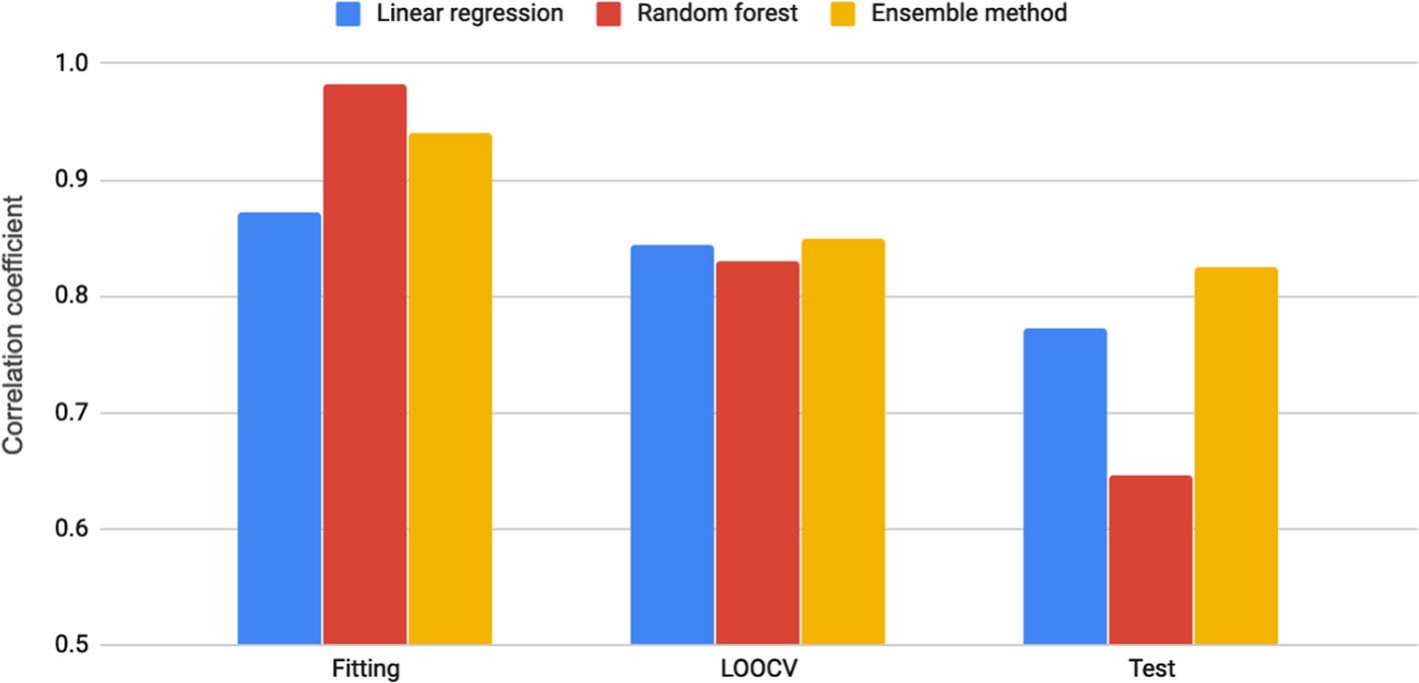
The comparison of linear, non-linear and ensemble models

The removal of useless features consisting of 103, 563, and 20 features with extreme values, scarcity, and small variation, respectively, lead to a dataset of 758 features for subsequent analysis. Since the dataset is quite small, we first compared the performance of two types of models including linear and non-linear models. The sequential forward feature selection algorithm was then applied to select informative features. The stopping criterion for feature selection is that the inclusion of an additional feature gives less than 1% correlation coefficient improvement. A total of seven features were selected for the highly interpretable linear regression models. The performance is shown in Fig. 1. For the prediction of ex vivo CI values, the developed linear regression model was underfitted with lower fitting performance. The corresponding correlation coefficient values are 0.871, 0.844, and 0.772 for fitting, LOOCV, and test, respectively. For the non-linear model, six features were selected for random forest regression. It is not surprising that the fitting performance is excellent with a correlation coefficient of 0.982. However, the correlation coefficient values of 0.829 and 0.647 for LOOCV and test, respectively, indicated that the model is overfitted. Since the linear model gave a stable performance and the non-linear model was good at fitting. Also, we argued that the informative feature set should be useful for both linear and non-linear models. The combination of both methods by voting regression could be a potential solution for the prediction of human placental barrier permeability.

### Ensemble model

**Fig. 2:**
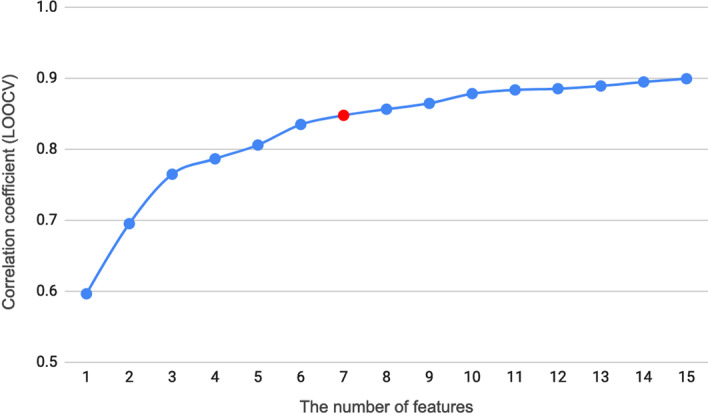
The feature selection process for the ensemble model. The red dot is the selected point for subsequent model development where the inclusion of one more feature does not make at least 1% improvement on the LOOCV correlation coefficient

The sequential forward feature selection algorithm identified 7 informative features of MLFER_BH, AATSC3v, AATSC2i, GATS1s, AATSC1c, MATS2i, and GATS5m for the ensemble model of voting regression. MLFER_BH represents the overall or summation solute hydrogen bond basicity [24]. The other six features belong to autocorrelation descriptors with different parameters [25]. The description of the 7 features is shown in Table 1 and the feature selection process is shown in Fig. 2. The fitting performance is between the linear and non-linear ones with a correlation coefficient of 0.94 (Fig. 1). The detailed fitting and LOOCV results are shown in Additional file 1: Table S1. Please note that the feature values shown in Additional file 1: Table S1 are z-score normalized values. Their corresponding mean and variance are shown in Additional file 1: Table S2.

**Table 1:** Description of the identified 7 informative features

Feature	Description	References
MLFER_BH	Overall or summation solute hydrogen bond basicity	Platts et al. [24]
AATSC3v	Average centered Broto-Moreau autocorrelation—lag 3/weighted by van der Waals volumes	Todeschini and Consonni [25]
AATSC2i	Average centered Broto-Moreau autocorrelation—lag 2/weighted by first ionization potential	
GATS1s	Geary autocorrelation—lag 1/weighted by I-state	
AATSC1c	Average centered Broto-Moreau autocorrelation—lag 1/weighted by charges	
MATS2i	Moran autocorrelation—lag 2/weighted by first ionization potential	
GATS5m	Geary autocorrelation—lag 5/weighted by mass

**Fig. 3:**
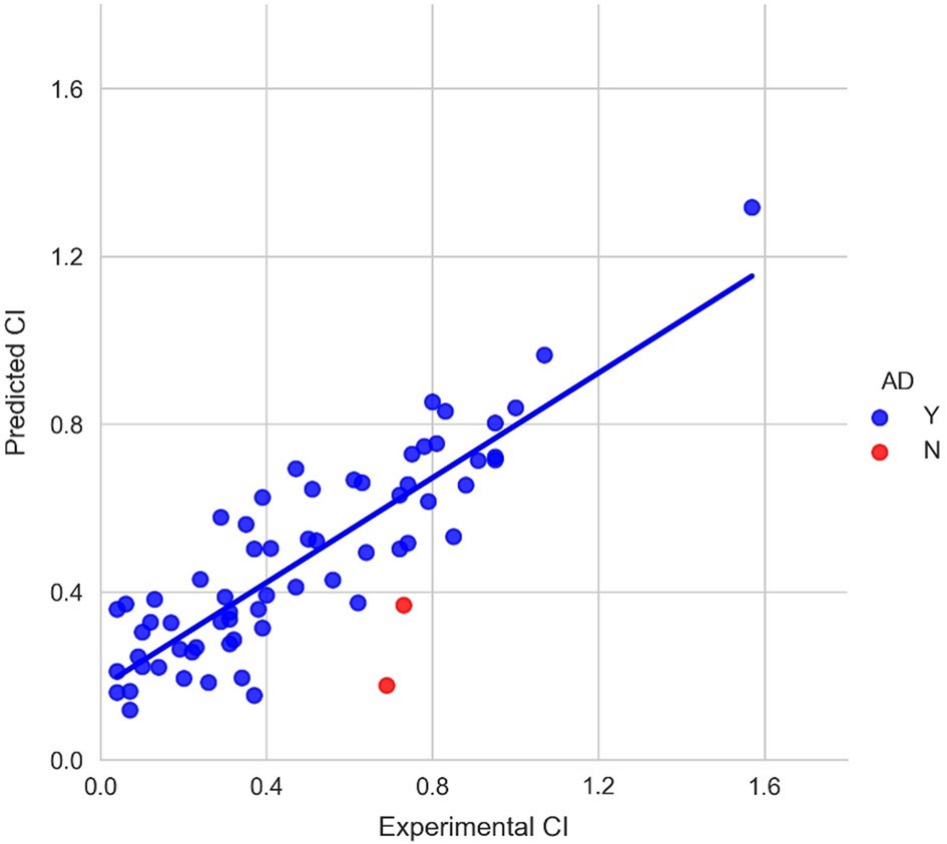
The comparison of experimental and predicted CI values for leave-one-out cross-validation. *AD*, applicability domain; Y, chemicals within the AD (blue dot); N, chemicals out of the AD

**Fig. 4:**
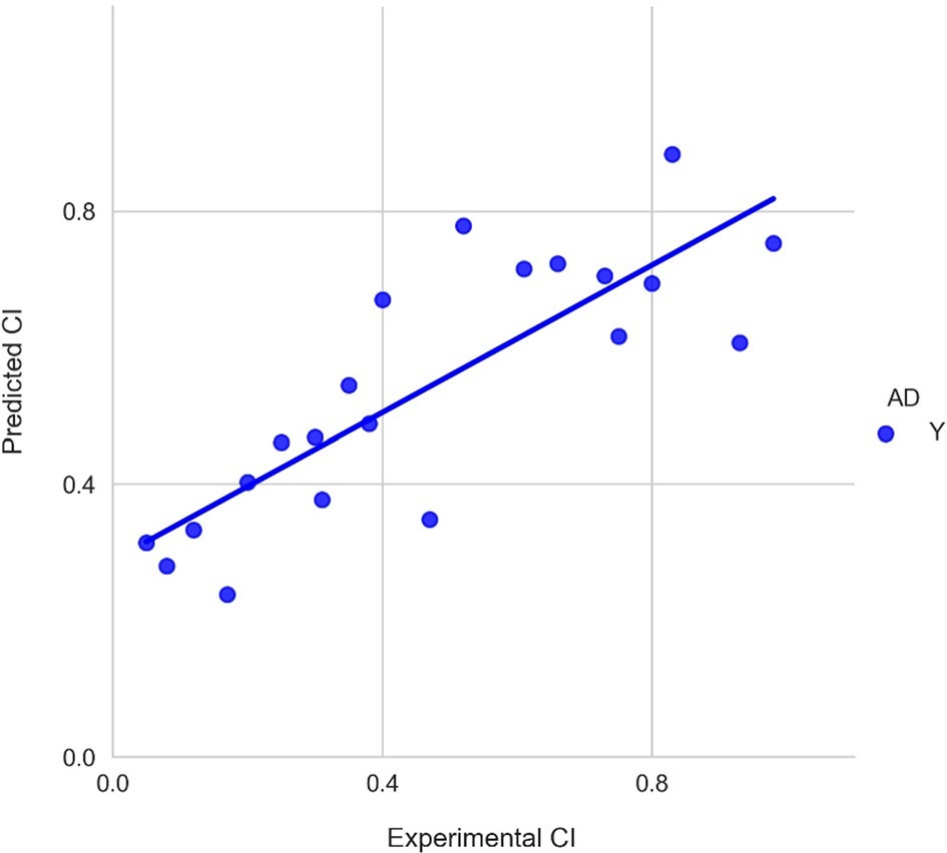
The comparison of experimental and predicted CI values for the test dataset. AD, applicability domain; Y, chemicals within the AD (blue dot)

The LOOCV performance was slightly improved compared to the linear and non-linear models with a correlation coefficient of 0.85 (Fig. 1). The results showed that different combinations of feature sets and algorithms performed similarly in LOOCV. The comparison of experimental and predicted CI values obtained from the LOOCV is shown in Fig. 3. While the LOOCV performance was only slightly improved, a much better test correlation coefficient value of 0.825 was obtained from the test dataset (Fig. 1). The small difference of LOOCV and test performance showed that there were no overfitting problems for the developed ensemble model. Figure 4 presents the plot of experimental and predicted CI values. Detailed prediction values on the test dataset are shown in Additional file 1: Table S3. Please note that the feature values shown in Additional file 1: Table S3 are z-score normalized values. The robust ensemble model could be useful for predicting ex vivo human placental barrier permeability.

### Adjustment of applicability domain

The determination of the applicability domain of models should be derived only from the training dataset and validated using the test dataset to avoid information leak issues [4, 5]. In this study, a useful decision tree-based method [1] was utilized to identify an exclusion rule set for chemicals out of the applicability domain based on the training dataset. Briefly, rules for predicting LOOCV errors were firstly derived using the CART algorithm. Subsequently, the rule with the highest predicted error was iteratively appended to the exclusion rule set until no significant improvement (< 1%) on the correlation coefficient was obtained. A total of two exclusion rules were determined to exclude chemicals out of the applicability domain, as shown in Additional file 1: Table S4. Please note that the exclusion rules are based on normalized feature values.

After the applicability domain adjustment, two chemicals of riboflavin and lopinavir in the training dataset were excluded based on rules of #1 and #2 (described in Additional file 1: Table S4), respectively. The exclusion of the two chemicals led to an improvement of correlation coefficient values of 0.952 and 0.887 for model fitting and LOOCV on the training dataset, respectively. The coverage of chemicals within the applicability domain is 96.97% (64/66). The application of the applicability domain identified no chemicals in the test dataset indicating that those test chemicals (coverage = 100%) could be reliably predicted.

### The comparison between the feature sets of three models

Since three feature sets were identified for the linear, non-linear, and ensemble methods, it is interesting to know the difference among the feature sets. Table 2 shows the selected features for the three models and their corresponding categories. The categorization is based on the document provided by the PaDEL-Descriptor software. The diverse descriptor types were selected for the linear regression model, while autocorrelation descriptors were preferred for random forest and ensemble models. All three types of models found that the autocorrelation descriptors were important for the prediction of ex vivo placental barrier permeability. Three features of AATSC3v, AATSC1c and MLFER_BH were identified to be useful for two types of models that might be more reliable predictors for ex vivo placental barrier permeability. It is worth noting that AATSC1c was also found useful for predicting in vivo logFM values for placental barrier permeability reported by our previous work [1]. Autocorrelation features could play important roles on the placental barrier permeability as they are recognized to be important for all three types of models.

**Table 2:** Comparison of the selected feature sets for the three types of models

Category	Linear regression	Random forest	Ensemble method
Autocorrelation	**AATSC3v**, ATSC2e, GATS8c,	**AATSC1c**, ATSC3v, GATS1s	**AATSC3v**, AATSC2i, GATS1s, **AATSC1c**, MATS2i, GATS5m
Barysz matrix		VE1_Dze, VE3_Dzm	
Detour matrix	VE1_Dt		
Extended topochemical atom	ETA_BetaP		
Molecular linear free energy relation		**MLFER_BH**	**MLFER_BH**
Path counts	R_TpiPCTPC		
Topological polar surface area	TopoPSA		

### Feature analysis showing the gap between ex vivo and in vivo conditions

Since informative features for ex vivo and in vivo datasets were identified by this study and previous studies [1, 2], respectively, it is interesting to know the difference between those two datasets for better interpretation of the predicted results. First, the correlation coefficients between the seven features from the ex vivo dataset and three features from in vivo dataset [2] were calculated to show the difference between the two conditions. As shown in Table 3, MLFER_BH is the only feature with good correlation coefficients of 0.762, 0.511 and 0.865 to all the three features of MW, hmax and TopoPSA for in vivo condition, respectively. In addition, MLFER_BH is the first feature selected by our ensemble model through stepwise feature selection showing that the in vivo information carried by MLFER_BH is very important and the proposed method was able to identify key features responsible for transplacental permeability. The absolute values of correlation coefficients for the other features are all less than 0.35 showing that the features may not be relevant to the in vivo transplacental permeability.

**Table 3:** Correlation coefficients between the informative features for in vivo and ex vivo datasets

Features	MW*	hmax*	TopoPSA*
MLFER_BH	0.762	0.511	0.865
AATSC3v	0.197	0.039	− 0.029
AATSC2i	0.043	0.187	0.019
GATS1s	− 0.321	− 0.144	− 0.082
AATSC1c	0.341	− 0.280	− 0.265
MATS2i	0.080	0.096	0.093
GATS5m	0.108	− 0.054	0.081

*Features for in vivo dataset were obtained from a previous study [2]

Second, MLFER_BH and the three features of MW, hmax and TopoPSA were utilized to train two separate models using the proposed ensemble method to know whether the features highly relevant to in vivo conditions are predictive of *ex viv*o condition. Similar moderate correlation coefficients of around 0.55 were obtained from the cross-validation of the two models showing that the features relevant to in vivo conditions are insufficient for predicting ex vivo transplacental permeability.

Third, the importance of the seven features identified in this study were analyzed to better understand the contribution of the features to ex vivo transplacental permeability. The feature rankings based on the coefficients of linear regression and feature importance of random forest and an overall feature ranking are shown in Table 4. While MLFER_BH was the first feature selected by our algorithm, its overall ranking is only in fifth place. In contrast, the feature of AATSC1c ranked first but with low correlations to the three features for in vivo conditions.

**Table 4:** The rank of linear regression and random forest of seven features

Feature	Coefficient of linear regression	Ranking by coefficient	Feature importance of random forest	Ranking by feature importance	Overall ranking
MLFER_BH	− 0.035	4	0.133	5	5
AATSC3v	− 0.039	3	0.159	3	2
AATSC2i	− 0.023	6	0.100	7	7
GATS1s	0.019	7	0.180	1	4
AATSC1c	0.045	2	0.170	2	1
MATS2i	0.072	1	0.101	6	3
GATS5m	− 0.029	5	0.157	4	5

Altogether, the results indicated that in vivo and ex vivo transplacental permeability of chemicals shared similar basic features that were represented by MLFER_BH in our model. However, the extrapolation from ex vivo to in vivo conditions should be carefully evaluated since the features highly relevant to in vivo conditions are not very predictive. It revealed the importance of this work by identifying the additional six autocorrelation features required for explaining the ex vivo conditions. Among the seven informative features, AATSC1c and AATSC3v are considered most important for modeling the ex vivo transplacental permeability that is consistent with the comparison analysis between the feature sets of three models.

### External test

The advantage of ex vivo placental barrier permeability prediction models over in vivo ones is that new data is expected to grow with time. In order to further test our model, we curated an additional external test dataset from six papers [26–31]. The seven features required for the prediction were firstly calculated based on PaDEL-Descriptor. A re-trained voting regression model based on 87 data consisting of the original training and test datasets was applied to predict the external test dataset. The observed, fitting, and LOOCV results of the new training model are shown in Additional file 1: Table S5 and Additional file 1: Figure S1. The corresponding rules for defining the applicability domain of the model are shown in Additional file 1: Table S6. Applicability domain assessment showed that all the 7 chemicals can be reliably predicted by the model, i.e. they are all within the applicability domain.

**Fig. 5:**
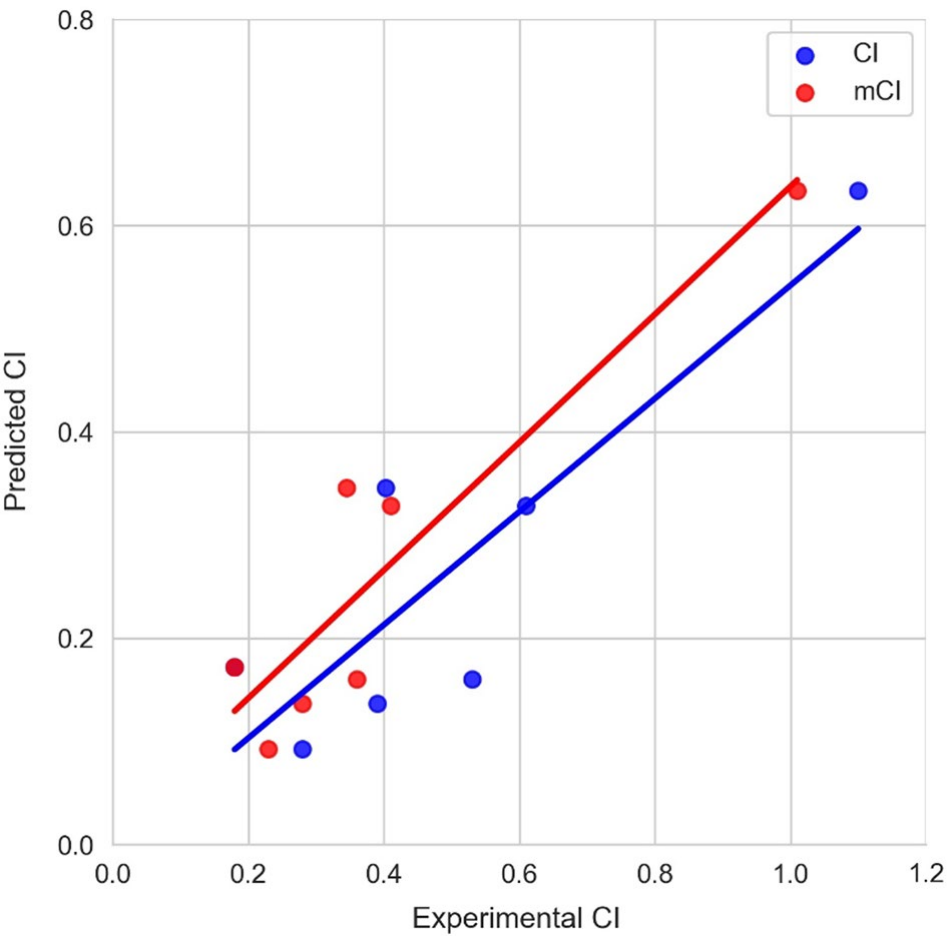
The comparison between CI and minimum CI values with predicted CI for the external test dataset

Detailed prediction results for the external test dataset are shown in Fig. 5 and Additional file 1: Table S7. The correlation coefficient and mean absolute error (MAE) values of the external test dataset were 0.879 and 0.232, respectively. While the performance is reasonably good, the results are based on the reported average CI values of several duplicates of ex vivo experiments curated from the literature. Considering the biological variation nature of the experiments, we further calculated the performance against the minimum CI (mCI) values within the variation. Results showed that the prediction made by our model well correlated with the mCI values with a correlation coefficient of 0.921 and MAE of 0.135, respectively. Among the seven chemicals, the absolute prediction errors for mCIs of bromocriptine and darunavir were less than 0.01 indicating a very accurate prediction. In contrast, the largest absolute error of 0.376 based on its mCI value has been made for bisphenol A. Nevertheless, the relative absolute error for bisphenol A is only 0.372 showing the usefulness of the prediction model. Altogether, the external test showed that the model is capable of producing reliable predictions for ex vivo human placental barrier permeability.

## Discussion

This work demonstrated that ensemble learning provides a more reliable prediction on ex vivo human placental barrier permeability. For small datasets, traditional methods relying on only a single linear/non-linear algorithm could overfit the dataset. Our evaluation results showed that single models derived from a small dataset could be unreliable. Moreover, the selected features are different for each algorithm due to the small number of samples. In this study, we argued that informative features should be useful for different types of algorithms. Our results showed that the selected features for the ensemble model are more predictive compared to the features for single algorithms. The selected features for the ensemble model are therefore considered more informative. The excellent test results on an additional test dataset confirmed the reliability of selected features and constructed model. Since a small dataset is a common issue for cheminformatics works, this study provides a potential solution for developing reliable models based on a limited number of data. The analysis of predictive features for in vivo and ex vivo transplacental permeability of chemicals revealed that additional features of autocorrelation descriptors should be taken into consideration for the extrapolation from ex vivo to in vivo conditions. Since in vivo data is not likely to grow, future works can be the development of models for mapping ex vivo results to in vivo permeability.

## Conclusion

Computational models are promising alternatives for reducing experimental testing costs. Valid models according to the OECD guideline can be utilized to filter out potential toxicants or identify safer drug candidates for further experimental validation. In this work, the performance of the three types of models were analyzed and led to the development of a final ensemble model for predicting ex vivo human placental barrier permeability. Among the seven important features for model development, MLFER_BH represents basic properties of in vivo transplacental permeability and autocorrelation descriptors are potential features for extrapolation from in vivo to ex vivo conditions. The identification of AATSC1c is also consistent with our previous study for predicting in vivo human placental barrier permeability [1]. In addition to the good performance derived from fitting, LOOCV, and independent test, an additional dataset consisting of seven chemicals curated from literature was applied to further test the predictive ability for unseen chemicals. An excellent correlation coefficient of 0.921 was obtained when considering the variation of biological experiments showing the usefulness of the developed model in the real world setting. Future works could be the development of strategies for integrating outputs from valid in vivo and ex vivo models for improving its performance. Also, our previous weight-of-evidence model for predicting reproductive and developmental toxicity [7] could be extended to incorporate placental barrier permeability for assessing embryo-fetal developmental toxicity.

## Supplementary Information

**Additional file 1: Table S1**. The features, experimental CI, predicted CI values, and AD of the training dataset. **Table S2**. The mean and variance values of the 7 selected informative features for the ensemble model. **Table S3**. The features, experimental CI, predicted CI values, and AD of the test dataset. **Table S4**. The rules for defining the applicability domain of the ensemble model based on the training dataset. **Table S5**. Details of the final prediction model based on all 87 chemicals. **Table S6**. The rules for defining the applicability domain of the final prediction model. **Table S7**. The features, experimental CI, predicted CI values, and AD of the external test dataset. **Figure S1**. The comparison of experimental and predicted CI values of 87 training dataset for leave-one-out cross-validation. Abbreviations: AD, applicability domain; Y, chemicals within the AD (blue dot); N, chemicals out of the AD (red dot).

## Data Availability

Data are available as Supplementary Files.

## Abbreviations

OECD: Organisation for Economic Co-operation and Development
ICH: International Council for Harmonisation of Technical Requirements for Pharmaceuticals for Human Use
CI: Clearance index
PCA: Principal component analysis
PLS: Partial least square
LOOCV: Leave-one-out cross-validation
CART: Classification and regression tree
MAE: Mean absolute error
mCI: Minimum clearance index

## References

[1] Wang C-C, Lin P, Chou C-Y, Wang S-S, Tung C-W (2020). Prediction of human fetal–maternal blood concentration ratio of chemicals. PeerJ.

[2] Takaku T, Nagahori H, Sogame Y, Takagi T (2015). Quantitative structure-activity relationship model for the fetal-maternal blood concentration ratio of chemicals in humans. Biol Pharm Bull.

[3] Grafmüller S, Manser P, Krug HF, Wick P, von Mandach U (2013). Determination of the transport rate of xenobiotics and nanomaterials across the placenta using the ex vivo human placental perfusion model. J Vis Exp JoVE.

[4] Tung C-W, Lin Y-H, Wang S-S (2019). Transfer learning for predicting human skin sensitizers. Arch Toxicol.

[5] Tung C-W, Wang C-C, Wang S-S (2018). Mechanism-informed read-across assessment of skin sensitizers based on SkinSensDB. Regul Toxicol Pharmacol.

[6] Borba JVB, Braga RC, Alves VM, Muratov EN, Kleinstreuer N, Tropsha A (2020). Pred-Skin: a web portal for accurate prediction of human skin sensitizers. Chem Res Toxicol.

[7] Tung C-W, Cheng H-J, Wang C-C, Wang S-S, Lin P (2020). Leveraging complementary computational models for prioritizing chemicals of developmental and reproductive toxicity concern: an example of food contact materials. Arch Toxicol.

[8] Amberg A, Beilke L, Bercu J, Bower D, Brigo A, Cross KP (2016). Principles and procedures for implementation of ICH M7 recommended (Q)SAR analyses. Regul Toxicol Pharmacol.

[9] Giaginis C, Zira A, Theocharis S, Tsantili-Kakoulidou A (2009). Application of quantitative structure–activity relationships for modeling drug and chemical transport across the human placenta barrier: a multivariate data analysis approach. J Appl Toxicol.

[10] OECD. Guidance document on the validation of (quantitative) structure-activity relationship models. 2014.

[11] Zhang Y-H, Xia Z-N, Yan L, Liu S-S (2015). Prediction of placental barrier permeability: a model based on partial least squares variable selection procedure. Molecules.

[12] Roy K, Kar S, Ambure P (2015). On a simple approach for determining applicability domain of QSAR models. Chemom Intell Lab Syst.

[13] Gramatica P (2007). Principles of QSAR models validation: internal and external. QSAR Comb Sci.

[14] Yap CW (2011). PaDEL-descriptor: an open source software to calculate molecular descriptors and fingerprints. J Comput Chem.

[15] Huang S-H, Tung C-W, Fülöp F, Li J-H (2015). Developing a QSAR model for hepatotoxicity screening of the active compounds in traditional Chinese medicines. Food Chem Toxicol Int J Publ Br Ind Biol Res Assoc.

[16] Tseng C-H, Tung C-W, Wu C-H, Tzeng C-C, Chen Y-H, Hwang T-L (2017). Discovery of Indeno[1,2-c]quinoline derivatives as potent dual antituberculosis and anti-inflammatory agents. Mol Basel Switz.

[17] Kreyszig E (1979). Advanced engineering mathematics.

[18] Tung C-W, Wu M-T, Chen Y-K, Wu C-C, Chen W-C, Li H-P (2013). Identification of biomarkers for esophageal squamous cell carcinoma using feature selection and decision tree methods. Sci World J.

[19] Wang C-C, Lin Y-C, Lin Y-C, Jhang S-R, Tung C-W (2017). Identification of informative features for predicting proinflammatory potentials of engine exhausts. Biomed Eng Online.

[20] Tung C-W (2013). Prediction of pupylation sites using the composition of k-spaced amino acid pairs. J Theor Biol.

[21] Breiman L (2001). Random forests. Mach Learn.

[22] Pedregosa F, Varoquaux G, Gramfort A, Michel V, Thirion B, Grisel O (2011). Scikit-learn: machine learning in python. J Mach Learn Res.

[23] Breiman L, Friedman JH, Olshen RA, Stone CJ. Classification and regression trees. 2017.

[24] Platts JA, Butina D, Abraham MH, Hersey A (1999). Estimation of molecular linear free energy relation descriptors using a group contribution approach. J Chem Inf Comput Sci.

[25] Todeschini R, Consonni V (2009). Molecular descriptors for chemoinformatics: volume I: alphabetical listing/volume II: appendices, references.

[26] Vinot C, Tréluyer J-M, Giraud C, Gavard L, Peytavin G, Mandelbrot L (2016). Bidirectional transfer of raltegravir in an ex vivo human cotyledon perfusion model. Antimicrob Agents Chemother.

[27] Mandelbrot L, Duro D, Belissa E, Peytavin G (2014). Placental transfer of darunavir in an ex vivo human cotyledon perfusion model. Antimicrob Agents Chemother.

[28] Mandelbrot L, Duro D, Belissa E, Peytavin G (2015). Placental transfer of rilpivirine in an ex vivo human cotyledon perfusion model. Antimicrob Agents Chemother.

[29] Mendes MDS, Hirt D, Vinot C, Valade E, Lui G, Pressiat C (2016). Prediction of human fetal pharmacokinetics using ex vivo human placenta perfusion studies and physiologically based models. Br J Clin Pharmacol.

[30] Balakrishnan B, Henare K, Thorstensen EB, Ponnampalam AP, Mitchell MD (2010). Transfer of bisphenol A across the human placenta. Am J Obstet Gynecol.

[31] Zheng Q, Zhou Q, Li J, Tian Y, Huang H, Yao Q (2019). Placental transfer of bromocriptine in an ex vivo human placental perfusion model. J Matern Fetal Neonatal Med.

